# Development of Spanish Broom and Flax Dressings with Glycyrrhetinic Acid-Loaded Films for Wound Healing: Characterization and Evaluation of Biological Properties

**DOI:** 10.3390/pharmaceutics13081192

**Published:** 2021-08-03

**Authors:** Angela Abruzzo, Concettina Cappadone, Valentina Sallustio, Giovanna Picone, Martina Rossi, Fiore Pasquale Nicoletta, Barbara Luppi, Federica Bigucci, Teresa Cerchiara

**Affiliations:** 1 Department of Pharmacy and Biotechnology, University of Bologna, Via San Donato 19/2, 40127 Bologna, Italy; angela.abruzzo2@unibo.it (A.A.); concettina.cappadone@unibo.it (C.C.); valentina.sallustio2@unibo.it (V.S.); giovanna.picone2@unibo.it (G.P.); martina.rossi12@unibo.it (M.R.); barbara.luppi@unibo.it (B.L.); federica.bigucci@unibo.it (F.B.); 2 Department of Pharmacy, Health and Nutritional Sciences, University of Calabria, 87036 Arcavacata, Rende, Italy; fiore.nicoletta@unical.it

**Keywords:** Spanish Broom, flax, wound dressings, glycyrrhetinic acid, polymeric films, sodium hyaluronate, human fibroblast SW1, biocompatibility, wound healing

## Abstract

The selection of an appropriate dressing for each type of wound is a very important procedure for a faster and more accurate healing process. So, the aim of this study was to develop innovative Spanish Broom and flax wound dressings, as alternatives to cotton used as control, with polymeric films containing glycyrrhetinic acid (GA) to promote wound-exudate absorption and the healing process. The different wound dressings were prepared by a solvent casting method, and characterized in terms of drug loading, water uptake, and in vitro release. Moreover, biological studies were performed to evaluate their biocompatibility and wound-healing efficacy. Comparing the developed wound dressings, Spanish Broom dressings with GA-loaded sodium hyaluronate film had the best functional properties, in terms of hydration ability and GA release. Moreover, they showed a good biocompatibility, determining a moderate induction of cell proliferation and no cytotoxicity. In addition, the wound-healing test revealed that the Spanish Broom dressings promoted cell migration, further facilitating the closure of the wound.

## 1. Introduction

Physical damage and diseases can alter the integrity of skin tissue, causing acute or chronic wounds that require effective management to avoid serious injures such as severe bleeding, bacterial infections, and inflammation, which threaten human health severely [1,2]. In general, the healing process is based on a cascade of events, including hemostasis, which occurs immediately after injury, followed by inflammation and proliferation, as well as remodeling of the tissue [3,4,5]. Hence, promotion of healing depends on the wound type and its associated pathological conditions, site of the wound, and the type of dressing materials [6], which can be generally classified into three classes based on their function: absorb exudate, and maintain or donate moisture [7]. Although traditional wound dressings (e.g., cotton bandages and gauze) represent the first type of wound dressings commonly in use, currently there are several commercially available products based on biopolymers that achieve optimal conditions for the promotion of healing process.

In this regard, the development of wound dressings based on new supporting materials remains a challenge. Spanish Broom and flax fabrics could be used as sustainable and biodegradable sources in substitution of cotton thanks to their availability, renewability, and cleaner and more resilient cultivation [8]. Although cotton is a soft, absorbent, and breathable natural fiber, widely used in textile and wound-care fields, its crops require a huge amount of water, pesticides, and fertilizer, causing a substantial strain on the environment [9]. Spanish Broom and flax fibers, as well as cotton, are composed of cellulose, a biocompatible and biodegradable polymer, useful in various applications such as scaffolds in tissue repair, wound dressing, artificial tissue/skin, controlled drug delivery, blood purification, and cell culture materials [10]. Our previous studies demonstrated that Spanish Broom dressings can be used for the delivery of different active molecules in the treatment of skin wounds [8,11,12,13]. Additionally, Spanish Broom, as well as flax, have presented interesting characteristics, such as a high hydrophilic nature and a capacity to absorb a large amount of water [14]. Glycyrrhetinic acid (GA) is a naturally pentacyclic triterpenoid extracted from *Glycyrrhiza glabra* L. liquorice roots [15] and has been chosen as bioactive molecule for wound treatment, taking into account its proven anti-inflammatory, antioxidant, and antimicrobial activities [16,17,18,19,20]. In order to exploit all of GA’s biological activities, there is an increasing interest in developing new formulations able to be directly applied to wounds. In recent years, nanoparticles, liposomes, and multifunctional nanofibers have been successfully investigated as drug carriers for wound healing [21]. In addition, polymeric films have been investigated to promote wound healing and to protect wounds against bacterial infections. Polymeric films are known to be one of the most popular choices for wound treatment, considering that they are simple, thin, and easy to prepare and apply [22]. Moreover, when in contact with wound exudates, they transform into a gel, creating a moist environment around the wound area, which is essential for effective chronic wound healing [23,24]. However, it has been reported that polymeric films alone are not recommended as dressings for wounds with excessive exudates due to low absorption capacity [25,26]. In this regard, a combination of different types of dressings; e.g., traditional wound dressings and polymeric films, could be used to heal wounds more quickly [27,28]

Therefore, in the present study we reported the development of innovative wound dressings able to join the different properties of the selected supporting materials and GA-loaded polymeric films to maintain a moist environment for wound healing and to improve the healing process [29]. As reported in the literature, various biopolymers such as chitosan (CH), sodium hyaluronate (HYA), sodium carboxymethylcellulose (CMC), and hydroxypropylmethylcellulose (HPMC) can facilitate the wound-healing process [30,31,32]. HYA, CMC, HPMC, and CH films on Spanish Broom and flax dressings were obtained by a solvent casting technique and characterized for their physico-chemical and functional properties, such as GA solid state, drug content, water uptake property, and ability to release GA. Cotton dressings with GA-loaded films were used as control. In addition, studies on cytotoxicity and cell proliferation were performed in human fibroblasts. Finally, the ability of the proposed wound dressings to promote cell migration, a crucial event for healing, was assessed.

## 2. Materials and Methods

### 2.1. Materials

Sodium hyaluronate (HYA; MW 800–1200 kDa) was sourced from Farmalabor (Canosa di Puglia, Italy). Sodium carboxymethylcellulose (CMC; MW 250 kDa, substitution degree 0.78) and hydroxypropylmethylcellulose (HPMC; Benecel™ K100M PHARM, MW 1000 kDa) were supplied by ACEF (Piacenza, Italy) and Ashland (Ashland, Switzerland), respectively. Low-viscosity chitosan from shrimp shells (CH; MW 150 kDa, deacetylation degree 96–98%), 18-β-glycyrrhetinic acid (GA; purity ≥ 97%), and all the solvents were purchased from Sigma-Aldrich (Milan, Italy). Flax dressings were obtained from Linificio e Canapificio S.r.l. (Villa D’Almè, Bergamo, Italy). Spanish Broom dressings were provided by Giuseppe Chidichimo of University of Calabria (Arcavacata di Rende, CS, Italy). Confiderm^®^ cotton gauzes were purchased from a local drugstore. Phosphate buffer solution at pH 7.4 (PBS) was prepared with the following composition: 2.38 g/L Na_2_HPO_4_·12 H_2_O, 0.19 g/L KH_2_PO_4_, and 8 g/L NaCl. Simulated wound fluid (SWF) was prepared with the following composition: 2 g/L bovine serum albumin, 23.40 g/L NaCl, 6.06 g/L Trizma base, and 2.20 g/L CaCl_2_ [22]. For GA determination, a phosphate buffer with 9.15 g/L Na_2_HPO_4_·12 H_2_O, adjusted to pH 7.0 with H_3_PO_4_ (pHmeter, MicroPH CRISON 2000, Carpi, Italy), was also prepared. The human fibroblast cell line SW1 was purchased from American Type Culture Collection (ATCC, Manassas, VA, USA). All reagents for cell culture were obtained from Sigma-Aldrich (St. Louis, MO, USA) if not otherwise specified, and were ultrapure grade. Dulbecco’s Modified Eagle Medium High glucose, Foetal Bovine Serum, and Dulbecco’s phosphate-buffered saline (DPBS) were from Euroclone (Pero, Milano, Italy). The alamarBlue reagent was from Thermo Fisher Scientific (Waltham, MA, USA). All plastic supports were from Falcon, Beckton Dickinson (Franklin Lakes, NJ, USA).

### 2.2. Preparation of Films on Cotton, Spanish Broom, and Flax Wound Dressings

Films on wound dressings were obtained by casting polymeric solutions on cotton, Spanish Broom, and flax dressings, and their subsequent desiccation by oven drying.

Firstly, polymeric solutions containing a plasticizer and GA were prepared. HYA, CMC, or HPMC were solubilized in water, while CH was solubilized in lactic acid solution (25 °C). Polymeric solutions were maintained under stirring at 200 rpm for 24 h at 25 °C in order to assure the complete dissolution of the polymer. After this time, propylene glycol, used as plasticizer [33], and GA, previously solubilized in the minimum amount of ethanol, were added. The addition of ethanol as co-solvent allowed us to enhance the solubility of GA, a molecule with low water solubility [34,35]. Solutions were left to stand at room temperature until all remaining air bubbles were eliminated.

Subsequently, 10 g of each polymeric solution were casted on cotton, Spanish Broom, and flax dressings (5.0 cm in diameter) placed in Petri dishes (5.2 cm in diameter). Finally, dressings were oven-dried at 70 °C for 8 h (Heating Oven FD series; Binder, Tuttlingen, Germany) and afterwards cut in discs of 10 mm in diameter and stored in a desiccator until use.

Preliminary studies were carried out to achieve composition of the optimal dressing. Specifically, different polymer (0.5–2% *w*/*w*), ethanol (5% and 10% *w*/*w*) and propylene glycol (0–13.65% *w*/*w*) amounts were tested. Thereafter, the composition of the polymeric solutions, reported in Table 1, was selected for the preparation of the final dressings. This composition allowed us to obtain polymeric films on the supporting materials that were easy to handle and remove from the Petri dish without damage, as well as GA solubilization and addition in wound dressings.

Loaded polymeric films without dressings were additionally obtained in order to evaluate the solid state of GA.

### 2.3. Characterization of Cotton, Spanish Broom, and Flax Wound Dressings

Dressing samples (discs 10 mm in diameter) were weighted, and the thickness was measured using an electronic digital caliper (Art. 1367 E 2900, Shanghai ShangErBo Import & Export Co., Shanghai, China). GA content was measured by putting the dressing (a disc 10 mm in diameter) in 5 mL of methanol. After 24 h, the dissolution medium was centrifuged at 14,500 rpm (12,400 RCF) for 15 min (Microspin 12, Highspeed Mini-Centrifuge, Biosan, Riga, Latvia), and the supernatant was analyzed by a previously reported HPLC method [8]. Briefly, the chromatographic system was composed of a Shimadzu (Milan, Italy) LC-10ATVP chromatographic pump and a Shimadzu SPD-10AVP UV–vis detector set at 250 nm. Separation was obtained on a Phenomenex (Torrance, CA, USA) Synergi Fusion-RP 80A (150 mm × 4.6 mm I.D., 5 µm) coupled to a Phenomenex (Torrance, CA, USA) SecurityGuard C18 guard cartridge (4 mm × 3.0 mm I.D., 5 µm). The mobile phase was a mixture of an aqueous phosphate buffer at pH 7.0 and acetonitrile (40:60, *v*/*v*). The flow rate was 0.4 mL/min, and manual injections were made using a Rheodyne 7125 injector with a 20 µL sample loop. Data processing was handled by means of a CromatoPlus computerized integration system (Shimadzu Italia, Milan, Italy). The calibration curve of concentration versus peak area ratio was plotted at a concentration range of 0.1–5 µg/mL, and a good linearity was found (R^2^ = 0.998).

### 2.4. Differential Scanning Calorimetry (DSC)

DSC experiments were performed on polymeric films (without dressings) in order to investigate the solid state of GA inside the formulations. Calorimetric measurements were conducted through a Netzsch DSC200 PC differential scanning calorimeter (Netzsch, Germany) using around 40 mg of each sample in aluminium crucibles (pan and pierced lid) under protective nitrogen flow (10 mL/min). The following setting parameters were used: temperature from 25 °C to 400 °C, heating rate of 10 °C/min, and a sensitivity ranging from 3.8 to 4.4 µV/mg, which allowed the determination of the small concentration of used GA.

### 2.5. In Vitro Water-Uptake Ability

Water-uptake ability was studied to investigate the hydration properties of the wound dressings with and without polymeric films. An accurately weighted dressing disc (10 mm in diameter) was placed on filter paper (3 cm × 3 cm) soaked in PBS or SWF and positioned on top of a sponge (12 cm × 9 cm × 2 cm) previously soaked in the hydration medium. The sponge was placed in a Petri dish filled with the same solution to a height of 0.5 cm [36]. Water uptake (WU) was determined as weight increase of the dressing for 360 min, according to the following equation:

WU (%) = (W_hdf_ − W_hf_ − W_dd_) × 100÷W_dd_

where W_hdf_ is the weight of hydrated dressings and wet paper filter, W_hf_ is the weight of wet paper filter, and W_dd_ is the weight of the dry dressings.

### 2.6. In Vitro Release Studies

GA release from the different wound dressings was determined by using Franz-type static glass diffusion cells (15 mm jacketed cell with a flat-ground joint and clear glass with a 12 mL receptor volume and a diffusion surface area of 1.77 cm^2^), equipped with a V6A Stirrer (PermeGearInc., Hellertown, PA, USA). A circular dressing disc (10 mm in diameter, drug content equal to 200 µg) was introduced in the donor compartment of Franz-type cell divided from the receptor compartment by means of a cellulose filter (MF-Millipore Membrane, mixed cellulose esters, pore size = 0.45 μm). The receptor compartment was filled with a PBS and ethanol mixture (7:3 *v*/*v*) or SWF. The system was thermostated at 32 °C, and at appropriate time intervals until 360 min, 200 μL aliquots were taken and replaced with the same volume of the fresh buffer. GA concentration was quantified in the receptor phase by HPLC. All experiments were performed under sink conditions (Cmax in medium < 10% Csaturation). To evaluate GA solubility in the media, an excess of GA was dispersed in 10 mL of PBS and ethanol mixture (7:3 *v*/*v*) or SWF under agitation (300 rpm) for 48 h at 25 °C. In order to remove the undissolved GA, the dispersions were centrifuged at 14,500 rpm (12,400 RCF) for 30 min, and afterwards, the supernatants were collected and filtered through a 0.22 µm pore-size cellulose acetate syringe filter (VWR International, Milan, Italy). Supernatants containing PBS and ethanol mixture (7:3 *v*/*v*) were diluted in methanol (1:9 *v*/*v*) and assayed for GA content by UV–vis (λ = 250 nm). SWF-based samples were diluted in the same solvent, and centrifuged (14,500 rpm, 12,400 RCF, 30 min) again to remove aggregates of albumin, which were spontaneously formed after the addition of methanol; the supernatants were analyzed by UV–vis. A calibration curve was obtained with a GA concentration range of 7–70 µg/mL, and a good linearity was found (R^2^ = 0.999).

The release of GA over time was reported as Mt/M0 (fractional amount), where Mt represents the cumulative amount of GA released at each time, and M0 the total GA mass into the dressing disc.

### 2.7. Preparation of Extracts

The wound dressings with the best properties in terms of hydration ability and GA release were selected to investigate the biological properties. A dressing sample (disc of 10 mm in diameter, drug content equal to 200 µg) was immersed in 5 mL of Dulbecco’s Modified Eagle Medium High Glucose at 25 °C for 6 h (after this time, the GA was completely released). The dressing was then removed from the medium, and the resulting suspension was centrifuged at 5000 rpm (1470 RCF) for 15 min (Microspin 12, Highspeed Mini-Centrifuge, Biosan, Riga, Latvia). The supernatant was isolated and filtered through 0.45 μm Millipore filters in order to obtain the final extract (GA final concentration around 36 µg/mL) in complete medium supplemented with 10% FBS, 2 mM L-Glutamine, 1000 units/mL penicillin, and 1 mg /mL streptomycin.

### 2.8. Cell Culture and Treatment

The human fibroblast SW1 cells were grown in DMEM High Glucose, supplemented with 10% foetal bovine serum, 2 mM L-Glutamine, 1000 units/mL penicillin, and 1 mg/mL streptomycin, at 37 °C in a 5% CO_2_/95% air humidified atmosphere. To evaluate the biological effect of the different wound dressings, the cells were seeded in multiwell plates and treated after 24 h for 1 and/or 2 days with the extracts prepared as described in Section 2.7, or with ethanol solution of GA (36 µg/mL).

### 2.9. Cell-Viability Assay

Cell viability was estimated using the colorimetric indicator AlamarBlue assay. This test allows the assessment of changes in metabolic activity reflecting the cell viability by using a resazurin-based reagent [37]. Briefly, after treatment at the indicated concentrations, the culture medium was exchanged with fresh solution containing 10% of AlamarBlue reagent and incubated for 4 h at 37 °C. Next, the medium was collected and the fluorescence was measured with a plate reader (EnSpire Multimode Plate Reader, Perkin-Elmer) by applying a λ_exc_ of 560 nm and λ_em_ of 590 nm. Finally, the percentage of AlamarBlue reduction was normalized to that of the basal medium by using the equation: F590 sample 100/F590 basal medium.

### 2.10. Cell-Growth Analysis

The effect of the extracts prepared as described in Section 2.6 and the GA sample on cell growth was monitored by incubating the cell culture plates into an Incucyte live-cell analysis system (Essen BioScience Ltd., Hertfordshire, UK). The plates were imagined every 3 h for 2 days, and the confluence was obtained through the IncuCyte ZOOM software.

### 2.11. Wound-Healing Assay by Quantitative Phase Imaging (QPI) Microscopy

To evaluate the healing ability of the selected wound dressings, SW1 fibroblasts were plated on 24-well culture dishes at a density of 3 × 10^4^ cells/well and incubated until the confluence. Cell monolayers were scratched manually with a p200 pipette tip and then washed twice with PBS to remove cellular debris. Prior to monitoring the wound healing, cells were treated with the extracts prepared as described in Section 2.6 and GA solution, using fresh medium for the controls.

QPI imaging was performed using a Livecyte microscope (Phase Focus, Sheffield, UK). QPI images were acquired every 120 min for 28 h with a 10× objective (0.25 NA), at 37 °C and 5% CO_2_. QPI data were analyzed using Cell Analysis Toolbox software (Phase Focus, Sheffield, UK) to evaluate the uncovered area after scratching.

### 2.12. Statistical Analysis

All results are shown as mean ± standard deviation (SD). The SD was calculated from the values of 3 independent experiments. Data from all experiments were analyzed using t-test or one-way ANOVA test and differences were deemed significant for * *p* < 0.05 and ** *p* < 0.01. All the graphs, calculations, and statistical analyses were performed using GraphPad Prism software version 8.0 for Windows (GraphPad Software, San Diego, CA, USA).

## 3. Results and Discussion

### 3.1. Characterization of Cotton, Spanish Broom, and Flax Wound Dressings

The solvent casting method allowed us to obtain films on dressings that were easy to handle and remove from the Petri dish without damage. The macroscopical observation highlighted that all the films were homogeneously distributed through the mesh of the dressings and transparent, excluding the HPMC film-based dressings, which showed an opalescent aspect.

Weight, thickness, and GA content of the different dressings were determined and are reported in Table 2. The weight of dressings increased in the following order: cotton < Spanish Broom < flax, as a consequence of the texture of the supporting materials. In fact, the weights of cotton, Spanish Broom, and flax without polymeric films were 2.2 ± 0.3 mg/cm^2^, 14.3 ± 0.8 mg/cm^2^, and 35.0 ± 1.8 mg/cm^2^, respectively. Taking into account the polymeric composition, no significant difference was observed between dressings with HYA, CMC, and HPMC films (*p* > 0.05), while dressings based on CH film were characterized by a higher weight with respect to the other polymeric films (*p* < 0.05), probably due to the presence of lactic acid.

The thickness of dressings increased in the following order: cotton < Spanish Broom < flax, as consequence of the thickness of the supporting material (the thicknesses of cotton, Spanish Broom, and flax without polymeric films were 0.34 ± 0.03 mm, 0.45 ± 0.03 mm, and 0.54 ± 0.02 mm, respectively). Moreover, no significant differences were observed in thickness values in the presence of the different polymers (*p* > 0.05).

Finally, as regards the GA content, for all the dressings based on HYA, CMC, and CH films, the experimental GA content was close to the theoretical one (255 µg/cm^2^) with low SD values, suggesting that the preparative method allowed us to obtain a homogenous GA distribution inside the dressings [38]. On the other side, in the case of HPMC-film-based dressings, the higher values of SD indicated that the GA was not evenly distributed throughout the film due to its low solubility in HPMC [39], also confirmed by the opalescent aspect that was visually observed. For this reason, HPMC-film-based dressings were excluded from the following evaluations.

### 3.2. Differential Scanning Calorimetry (DSC)

**Figure 1:**
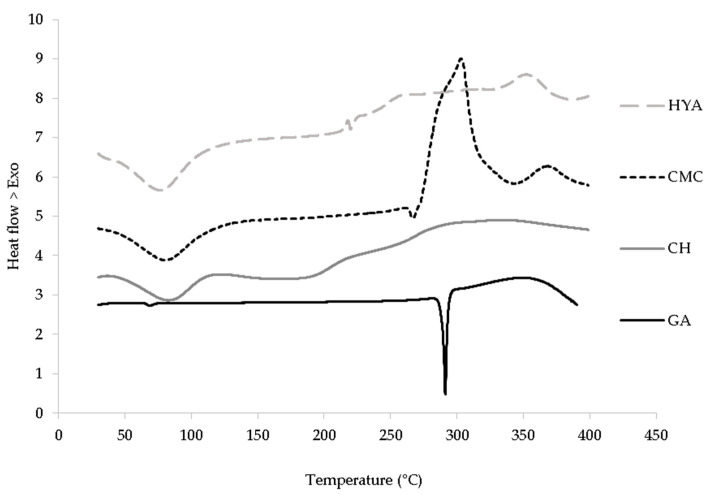
DSC thermograms of GA and GA-loaded polymeric films.

Figure 1 shows the DSC profile of GA overlapping with the profiles of loaded films based on HYA, CMC, and CH. GA showed a peak around 299.5 °C, corresponding to its melting point, in agreement with the literature [40]. The thermograms of all the films showed one endothermic peak around 76–83 °C that was attributed the loss of water molecules, and an exothermic peak beyond 200 °C due to the polymer decomposition. Moreover, the characteristic melting peak of GA was absent, indicating that the solvent casting method caused GA to transition from a crystalline to an amorphous state, which positively affected GA solubility [41].

### 3.3. In Vitro Water-Uptake Ability

The water-uptake ability of dressings represents a crucial property for wound-healing treatment. Particularly, it is responsible for the dissolution and release of the bioactive molecules, and it is essential for the treatment of skin wounds, especially for those producing a significant amount of exudate. In fact, the formulation ability to absorb biological fluids can contribute to maintain a moist wound environment, which is required to prevent tissue dehydration and promote tissue epithelialization and angiogenesis, and clearance of dead tissue [42]. Additionally, adequate dressing hydration ability can avoid the accumulation of excess exudate on the wound, which can slow wound healing and cause skin maceration [32].

**Figure 2:**
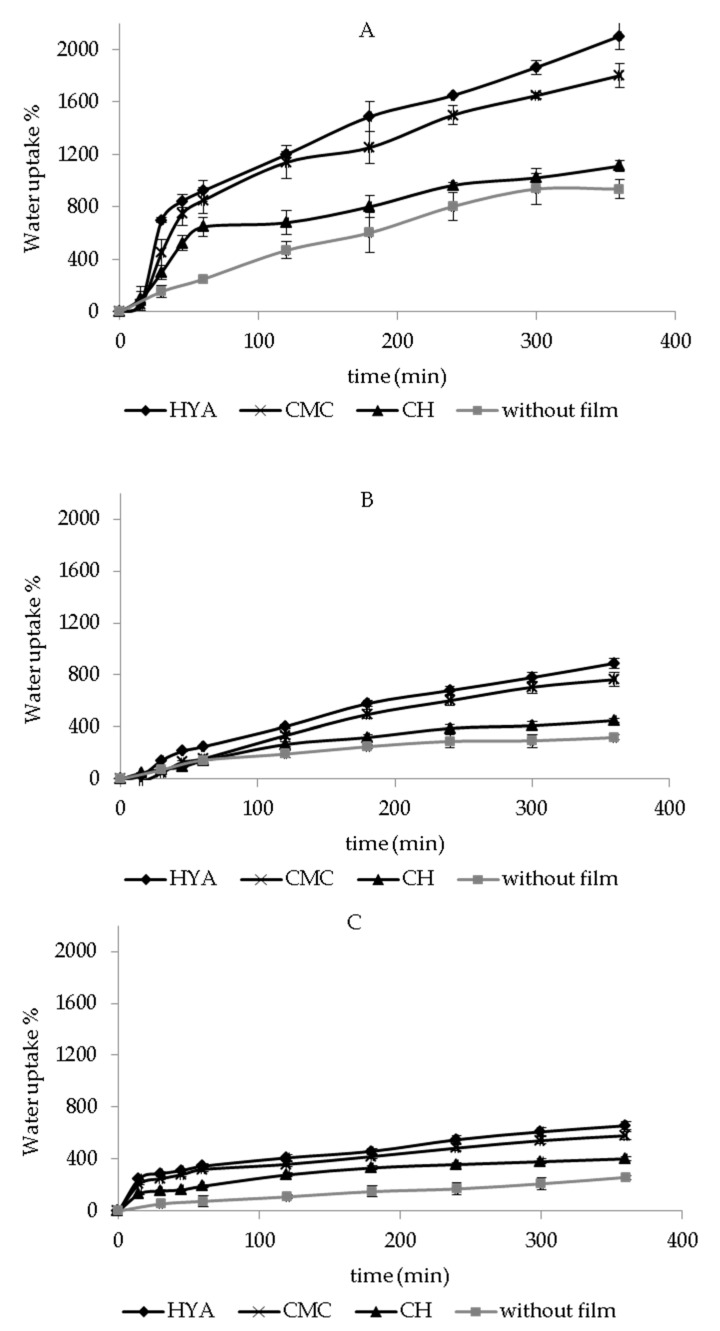
In vitro water-uptake profiles of cotton (**A**), Spanish Broom (**B**), and flax (**C**) wound dressings, with and without polymeric films, in PBS (mean ± SD, *n* = 3).

Figure 2 shows the in vitro water-uptake profiles of each dressing, with and without polymeric films, after the contact with PBS. The hydration abilities of cotton, Spanish Broom, and flax without polymeric films were measured, and after 6 h the water-uptake values were equal to 936 ± 70%, 320 ± 18%, and 259 ± 15%, respectively. The obtained results highlighted that the water-uptake ability increased in the following order: flax < Spanish Broom < cotton, as a consequence of the higher fineness of cotton and Spanish Broom fibers compared to flax, due to the physical-chemical process used to extract the fibers [14]. However, the fineness variation can be attributed to the different number of cells in the bundle and quality of fibers that can be extracted using different methods that allow the extraction of cellulose and non-cellulose compounds.

Moreover, this finding could be also related to the higher thickness of flax and Spanish Broom with respect to cotton, which limited the contact between the dressing and the sponge soaked with the medium, thus decreasing the water diffusion inside the dressings [43].

In the presence of polymeric films, an increase in water-uptake ability was observed (*p* < 0.05), thus demonstrating the ability of polymers to promote the entry of additional water inside the wound dressings. Although it has been reported that polymeric films are not recommended as dressings for wounds with excessive exudates due to low absorption capacity [25,26], our results demonstrated that the hydration ability of the final dressings could be improved by combining the selected supporting material with the polymeric films. Moreover, water-uptake ability of the dressings was also influenced by polymeric composition and increased in the following order: CH < CMC < HYA. These results could be attributed to the chemical structure of the polymers, as well as to their ionization in PBS [44]. The highest hydration ability of HYA-film-based dressings could be due to their polymeric hydrophilic nature, as well as to the presence of a high density of charged carboxyl groups (pKa = 2.9) on HYA chains able to promote the water uptake [43,45]. A slight reduction in water-uptake ability was observed in the presence of CMC films with respect to the HYA-film-based dressings (*p* < 0.05), probably due to the presence of a lower density of carboxyl charged groups (pka = 4.3), in agreement with our previous results [43]. Finally, the low ionization degree of aminic groups (pKa = 6.3) of CH led to the lowest water-uptake ability of CH-film-based dressings [46]. Moreover, a possible interaction between GA carboxyl groups and CH aminic groups [47] could also reduce the amount of charged groups and consequently decrease the water uptake.

**Figure 3:**
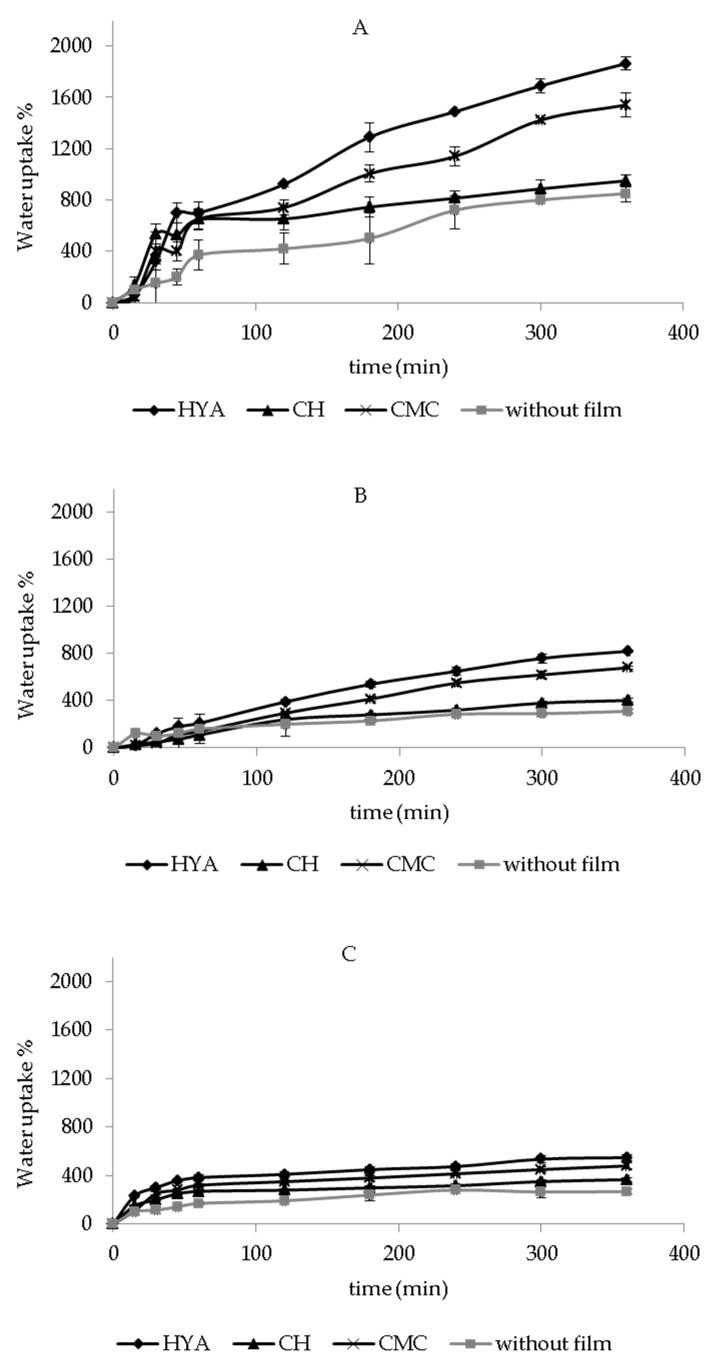
In vitro water-uptake profiles of cotton (**A**), Spanish Broom (**B**), and flax (**C**) wound dressings with and without polymeric films in SWF (mean ± SD, *n* = 3).

Figure 3 reports the in vitro water-uptake profiles of cotton, Spanish Broom, and flax wound dressings, with and without polymeric films, in SWF. The same trend of hydration behavior observed in PBS was also obtained in SWF. Furthermore, as can be seen, for dressings with polymeric films, a slight reduction in water uptake % values was observed with respect to PBS (*p* < 0.05), probably as a consequence of the interaction between SWF components and the polymers, which limited the presence of charged groups and consequently the entry of water [42,48]. Regarding dressings without polymeric films, no significant difference was observed between water-uptake values obtained in PBS and SWF (*p* > 0.05).

Taking into account these results, between flax and Spanish Broom, the latter showed the highest hydration ability, and could be proposed as an alternative to cotton dressings. Moreover, HYA-film-based dressings, bearing the highest hydration ability, could be considered promising, and could be proposed as potential dressing candidates for wound-healing treatment.

### 3.4. In Vitro Release Studies

**Figure 4:**
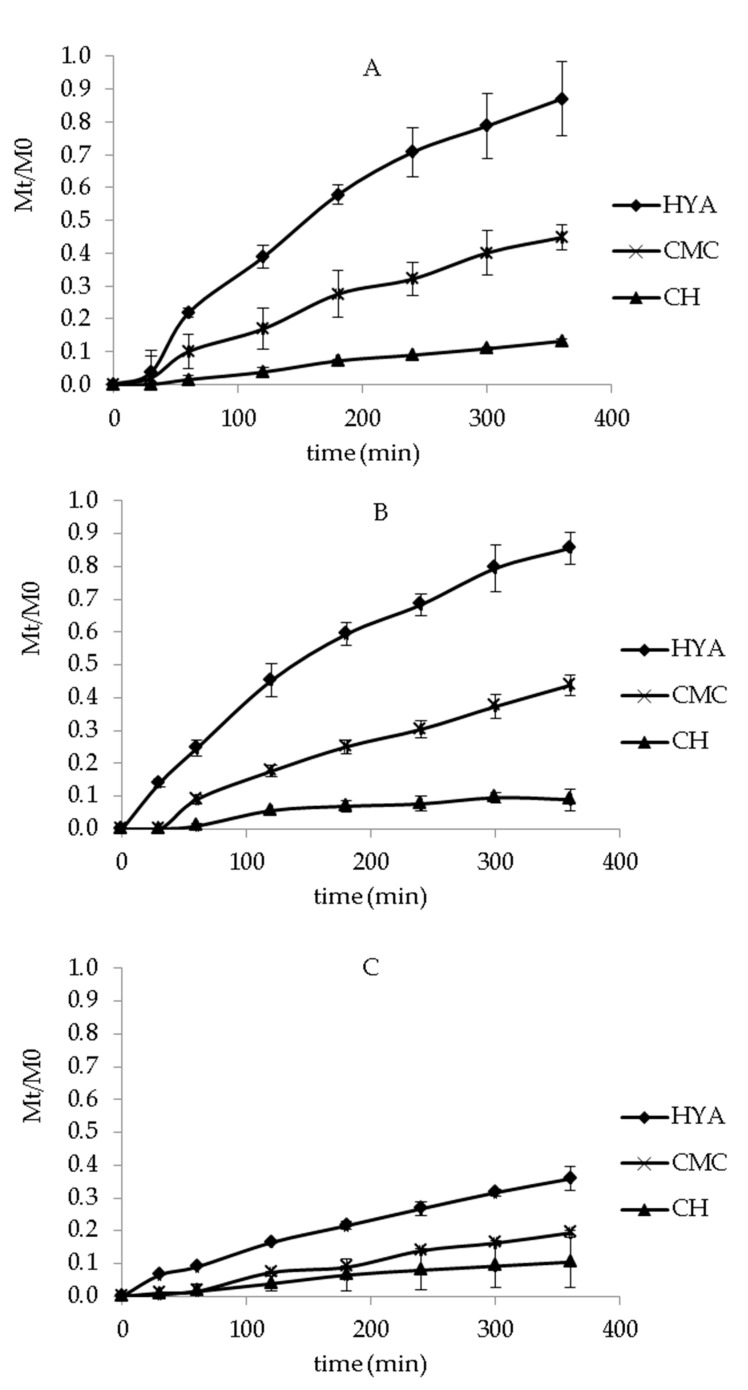
In vitro release profiles of GA from cotton (**A**), Spanish Broom (**B**), and flax (**C**) wound dressings in PBS/EtOH 7:3 *v*/*v* (mean ± SD, *n* = 3).

In vitro release tests were performed in order to evaluate dressings’ ability to release GA over the time. All experiments were conducted under sink conditions (Cmax in medium < 10% Csaturation). GA solubility in PBS and ethanol mixture (7:3 *v*/*v*) and SWF were 0.795 ± 0.042 mg/mL and 0.172 ± 0.002 mg/mL, respectively. Different factors, such as hydration ability, relaxation of the polymer chains, formation of viscous gel, and dissolution and diffusion of active molecules through the rehydrated formulation and the supporting material, could be involved in the release mechanism [49]. Figure 4 shows the in vitro release profiles of GA from the different dressings in PBS/ ethanol (7:3 *v*/*v*). No significant difference was observed between the amount of GA released from Spanish Broom and cotton (*p* > 0.05). Moreover, flax dressings provided the release of a lower amount of GA with respect to Spanish Broom and cotton (*p* < 0.05). This behavior was probably related to the hydration property of the supporting materials, and generally, a lower water uptake implied a lower release of GA from the dressings. In fact, as previously described, flax dressings were characterized by low fineness that limited the entry of water and consequently reduced the amount of GA released over the time. The release of GA from the dressings was also influenced by the polymeric composition, and increased in the following order: CH < CMC < HYA. Again, this result probably can be attributed to the different hydration abilities of the polymeric films. CH-film-based dressings showed the release of the lowest amount of GA over time (*p* > 0.05). This result can be explained by the low water-uptake ability together with the possible interaction between the GA carboxyl groups and CH aminic groups [47], limiting GA release. For CMC- and HYA-film-based dressings, the profiles also highlighted that GA was gradually released over 360 min. Considering that the first 12–48 h are decisive during the wound-healing process [44], this result represented a particularly important aspect in order to assure a continuous effect of GA and potentially reduce the frequency of dressing replacement, thus increasing patient compliance. Finally, among all the tested polymers, HYA allowed us to obtain the highest release of GA, reaching 87.1 ± 1.1%, 85.6 ± 0.5%, and 35.8 ± 8.6% of the total amount of GA for cotton, Spanish Broom and flax, respectively, after 360 min.

Taking into account water uptake and in vitro release results, HYA-film-based dressings were selected for further studies. In fact, HYA-film-based dressings showed the best hydration ability and provided the release of the highest amount of GA. Moreover, the importance of HYA in wound repair and tissue regeneration is well known [24]. In fact, it has been reported that HYA is involved in wound healing increasing keratinocyte migration and proliferation, and facilitating transport of nutrients and waste products [50,51].

**Figure 5:**
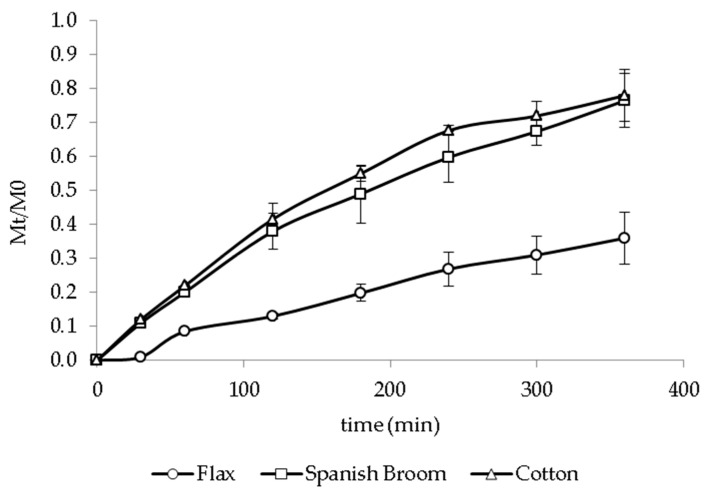
In vitro release profiles of GA from HYA-film-based cotton, Spanish Broom, and flax wound dressings in SWF (mean ± SD, *n* = 3).

Figure 5 shows the in vitro release profiles of GA from HYA-film-based dressings in SWF. As can be seen, no significant difference was observed between the amount of released GA in PBS and SWF (*p* > 0.05), despite the lower water-uptake ability measured in SWF. This behavior probably could be correlated with SWF composition, which can improve GA dissolution, and consequently its release.

### 3.5. Biological Studies

In order to study the cytotoxicity of the selected wound dressings, their effect on cell viability was evaluated with an alamarBlue assay. As the experimental model, a human fibroblast cell line was chosen, this being the kind of cell directly involved in the proliferative phase of the wound-healing process [52].

**Figure 6:**
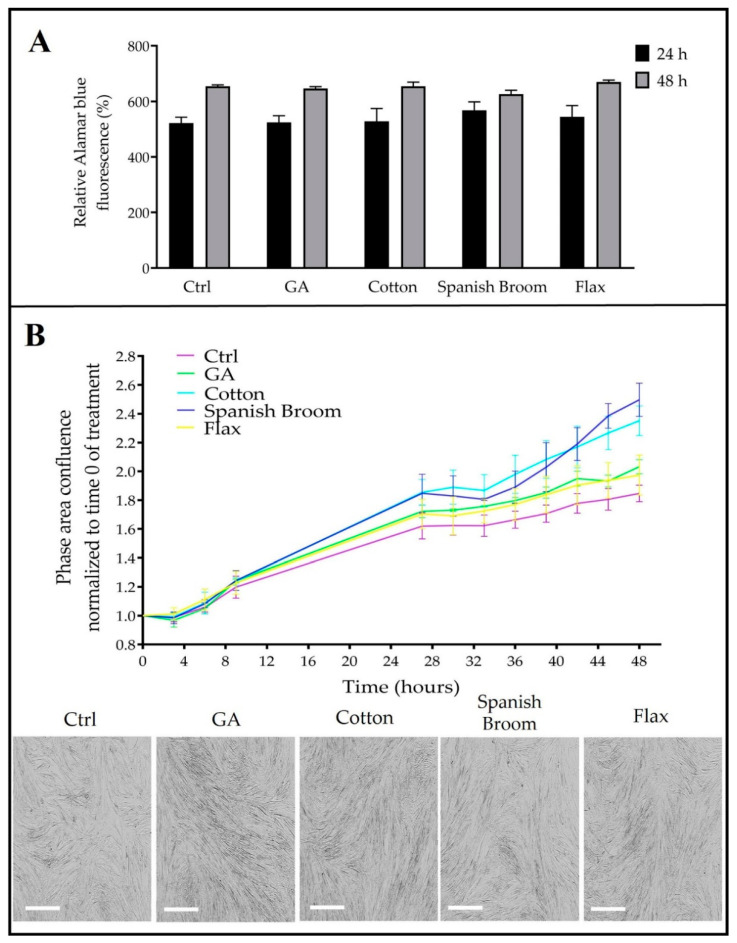
Effect of GA and extracts on human fibroblast viability. (**A**) Percent reduction of alamarBlue after 24 and 48 h of treatment. (**B**) Monitoring of phase-area confluence of cell culture for 48 h, obtained with an IncuCyte system instrument, and microphotographs of the cells. The phase-area confluence in cotton- and Spanish Broom-treated cells was increased at 48 h with respect to the control. Bar = 400 µm. One-way ANOVA test: *p* < 0.05 (cotton), *p* < 0.01 (Spanish Broom).

After seeding, cells were treated with extracts obtained as described in Section 2.7 or with ethanol solution of GA (see Section 2.8). The results reported in Figure 6A show that no cytotoxic effect was observed either at 24 or 48 h of treatment: the percentage of fluorescence, which is proportional to metabolically active cells, overlapped the controls in each sample. Moreover, we monitored the confluence of treated samples by means of an IncuCyte instrument, as it allowed for automated data acquisition of phase-contrast images within the cell culture incubator. Interestingly, all treatments increased the cell density with respect to the controls, although only the extracts from cotton and Spanish Broom dressings returned statistically significant values at 48 h. In particular, the values of the phase area confluence ratio for extracts derived from cotton and Spanish Broom dressings were 2.24 ± 0.19 and 2.28 ± 0.28 respectively, versus the control, which was equal to 1.78 ± 0.14 (Figure 6B).

It is worth noting that cells exhibited a regular morphology after treatment, confirming the biocompatibility of the proposed dressings and suggesting the eligibility of the cotton and Spanish Broom wound dressings. Considering that GA and HYA were present in all extracts, it is conceivable that the chemical composition of the two highest-performing wound dressings could promote the growth of dermal cells and the closure of the wound.

**Figure 7:**
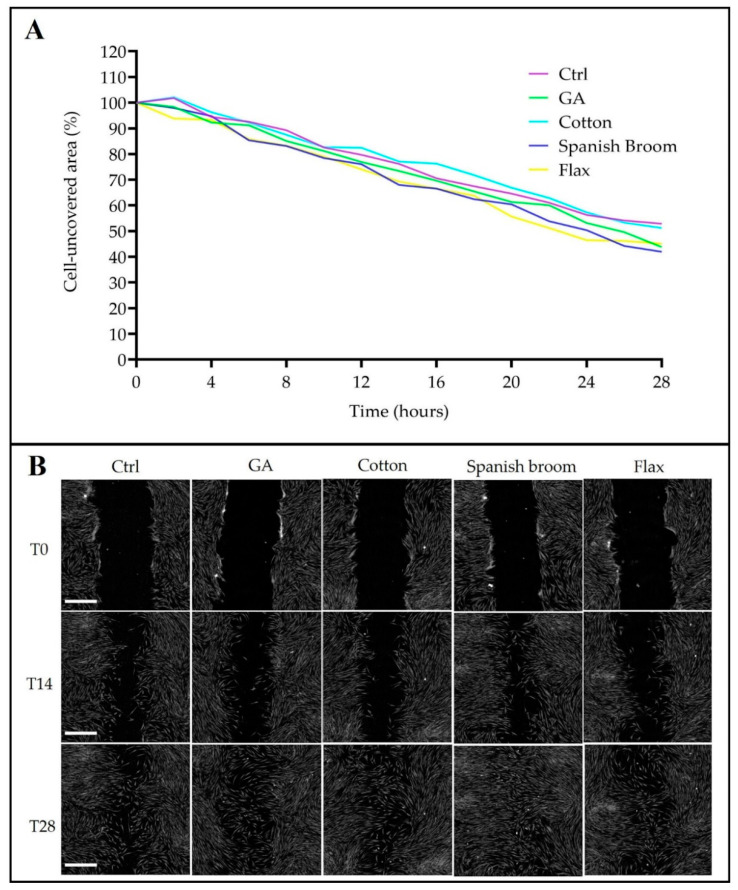
Wound-healing effect of GA and extracts on SW1 human fibroblasts. (**A**) Monitoring of uncovered area of cell culture for 28 h. The residual wound area was calculated as the ratio of the wound area post-treatment and the original wound area at time 0, taken as 100% (*n* = 4). The percentage of uncovered area was reduced by treatment with GA and Spanish Broom dressings at 28 h. One-way ANOVA test: *p* < 0.05. (**B**) Quantitative phase imaging showing the covered area at 0, 14, and 28 h after wounding. Each image is representative of a scratch assay of the four experimental cell groups. Bar = 500 µm.

It has been known that the ability of cells to migrate is essential for many physiological processes, including wound repair [53]. Hence, to deeply investigate the capability of the dressings to promote the healing process, we performed a scratch test on human fibroblasts. In particular, we monitored the uncovered area for 28 h post-treatment, as the ratio between the wound area and the original wound area at time 0 (Figure 7A). Although at the end point (28 h) the entire area seemed to be equally covered in all samples, it was possible to observe that at an intermediate stage of the migration process, there were some visibly appreciable differences (Figure 7B). Nevertheless, also after 28 h, the differences were statistically confirmed. In fact, the percentage of the uncovered area was 52.8% for controls, 51.2% for cotton dressings, 45.1% for flax dressings, 43.7% for GA, and 41.9% for Spanish Broom dressings, respectively. These results highlighted the suitability of the Spanish Broom dressings, which exhibited the highest induction to close the wounds, which was well evident, especially in a short time. This effect could depend on a synergy between the GA and the chemical composition of the Spanish Broom dressings. However, to corroborate this hypothesis, a qualitative analysis of the extract should be carried out in future studies. It was also very interesting to note the wound-healing activity shown by the GA alone, apparently not in line with the previous results on confluence. On the other hand, it is well known that the wound-healing assay describes cell migration. which represents a key event for tissue repair, not always related to cell proliferation.

## 4. Conclusions

The development of innovative wound dressings is based on the selection of appropriate biomaterials that promote the healing process. In this regard, Spanish Broom and flax dressings with polymeric (HYA, HPMC, CMC, and CH) films containing GA were prepared by a solvent casting method to promote the healing process and wound-exudate absorption, and were proposed as alternatives to cotton dressings, which were used as the control. Comparing all the wound dressings, Spanish Broom dressings based on HYA film showed the best functional properties in terms of hydration ability and GA release. Biological studies showed a good biocompatibility for the new proposed dressings, as the treatment of human fibroblasts with extracts did not impair their regular growth and morphology. Interestingly, the cotton and Spanish Broom dressings exhibited a moderate induction of cell proliferation. Nevertheless, the best-performing supporting material resulted from the Spanish Broom dressings because they were able to induce cell proliferation as well as cell migration, thus favouring the closure of the wound. Taking into account these results, the Spanish Broom dressings could represent a valid alternative to cotton dressings for the treatment of skin wounds.

## Figures and Tables

**Table 1:** Composition (% *w*/*w*) of polymeric solutions used for the preparation of wound dressings.

Components of Polymeric Solutions	HYA	CMC	HPMC	CH
Polymer	1	1	1	1
GA	0.05	0.05	0.05	0.05
Ethanol	10	10	10	10
Propylene glycol	13.65	13.65	13.65	13.65
Lactic acid	0	0	0	0.9
Water	75.3	75.3	75.3	74.4

**Table 2:** Weight, thickness, and GA content of wound dressings (mean ± SD, *n* = 5).

	Cotton			Spanish Broom			Flax		
	Weight (mg/cm^2^)	Thickness (mm)	Drug Content (µg/cm^2^)	Weight (mg/cm^2^)	Thickness (mm)	Drug Content (µg/cm^2^)	Weight (mg/cm^2^)	Thickness (mm)	Drug Content (µg/cm^2^)
HYA	7.2 ± 0.4	0.39 ± 0.03	244.8 ± 10.0	18.7 ± 0.9	0.46 ± 0.02	232.4 ± 16.1	39.1 ± 1.8	0.54 ± 0.02	257.7 ± 11.2
CMC	7.6 ± 0.3	0.34 ± 0.03	246.0 ± 11.8	19.7 ± 0.5	0.47 ± 0.02	263.9 ± 18.8	42.0 ± 1.9	0.54 ± 0.03	282.2 ± 13.5
HPMC	5.0 ± 0.2	0.39 ± 0.02	143.1 ± 85.7	19.2 ± 0.5	0.44 ± 0.02	147.5 ± 50.0	41.3 ± 1.2	0.57 ± 0.02	158.9 ± 56.9
CH	11.7 ± 0.4	0.38 ± 0.03	266.2 ± 18.8	25.4 ± 1.5	0.45 ± 0.03	237.9 ± 17.3	46.9 ± 1.2	0.53 ± 0.03	263.9 ± 16.6

## Data Availability

Data are contained within the article.
